# Editorial: Host factors in hepatitis B virus: mechanistic insights and implications for interferon therapy

**DOI:** 10.3389/fimmu.2026.1797907

**Published:** 2026-03-05

**Authors:** Hongxiao Song, Yongye Huang, Chunfeng Li, Qingfei Xiao, Guangyun Tan

**Affiliations:** 1 Department of Hepatology, Infectious Diseases and Pathogen Biology Center, Institute of Translational Medicine, The First Hospital of Jilin University, Changchun, Jilin, China; 2 College of Life and Health Sciences, Northeastern University, Shenyang, China; 3 Gilead Sciences, Inc., Foster City, CA, United States; 4 Department of Nephrology, The First Hospital of Jilin University, Changchun, China

**Keywords:** hepatitis B core protein (HBc), hepatitis B virus (HBV), host cellular factors, interferon therapy, metabolic regulation

Chronic hepatitis B virus (HBV) infection remains an urgent global health challenge, affecting hundreds of millions of individuals worldwide and markedly increasing the risk of cirrhosis and hepatocellular carcinoma. Although nucleos(t)ide analogs effectively suppress viral replication, their inability to achieve complete viral clearance highlights the necessity of therapeutic strategies that target host–virus interactions throughout the HBV life cycle. Among available treatment options, interferon (IFN) therapy is distinguished by its dual direct antiviral and immune-regulatory activities; however, its clinical application is limited by heterogeneous patient responses and frequent treatment-associated adverse effects. Understanding how host cellular factors regulate HBV replication and how interferon reshapes these interactions is therefore central to improving therapeutic efficacy and tolerability.

A defining theme of this Research Topic is that HBV persistence depends on the coordinated engagement of host-derived cellular machinery across multiple stages of the viral life cycle. Viral proteins serve as critical interfaces in this process, and among them, the hepatitis B core protein (HBc) emerges as a central regulatory hub (1). One comprehensive review in this Research Topic highlights HBc as more than a structural component of nucleocapsid assembly, emphasizing its multifunctional roles in coordinating host transcriptional regulation, intracellular trafficking, and innate and adaptive immune responses. By integrating recent advances in HBc structure, post-translational modification, and host protein interactions, this work positions HBc as a key determinant of viral persistence and a promising target for host-directed antiviral strategies.

Beyond viral hubs, mechanistic studies in this Research Topic underscore the importance of host metabolic and transcriptional regulators in controlling HBV infection (2). Original research reveals that sterol regulatory element-binding protein 2 (SREBP2), a master regulator of cholesterol biosynthesis, can directly influence HBV transcriptional activity through its interaction with viral components. These findings provide a mechanistic link between host metabolic state and viral replication, illustrating how cellular metabolic pathways can exert direct antiviral effects.

Interferon signaling intersects extensively with these host–virus interactions. Rather than acting solely through direct viral inhibition, IFN reshapes the intracellular environment by inducing antiviral restriction factors, modulating innate immune sensing pathways, and reprogramming cellular metabolism (3). Reviews included in this Research Topic emphasize the pleiotropic nature of interferon responses, highlighting their roles in coordinating innate and adaptive immunity while also revealing the intrinsic vulnerability of these pathways to dysregulation—providing a biological explanation for the variability of IFN treatment responses.

Importantly, interferon-associated adverse effects represent a unifying and clinically relevant concern across the studies presented here. IFN therapy is frequently accompanied by systemic side effects, including flu-like symptoms, hematologic toxicity, neuropsychiatric disturbances, and metabolic dysregulation, which often compromise treatment adherence and duration. Clinical investigations in this Research Topic demonstrate that host background conditions critically modulate this balance between antiviral benefit and toxicity (4). In particular, research examining patients receiving pegylated interferon therapy shows that metabolic dysfunction-associated steatotic liver disease (MASLD) significantly impairs antiviral efficacy while exacerbating liver-related risk, underscoring how host metabolic context can simultaneously blunt therapeutic response and amplify interferon-related adverse effects.

In addition to baseline host characteristics, treatment duration emerges as a key determinant of interferon-related burden. Prolonged IFN exposure increases the likelihood of immune-mediated inflammation and systemic toxicity without guaranteeing proportional antiviral benefit (5). Studies in this Research Topic demonstrate that quantitative host markers—most notably baseline and early on-treatment hepatitis B surface antigen (HBsAg) levels—can inform the duration required to achieve clinical response, supporting a precision-treatment paradigm that minimizes unnecessary IFN exposure while preserving antiviral efficacy.

Collectively, the contributions in this Research Topic provide an integrated framework for understanding how host cellular factors regulate HBV infection and how interferon therapy reshapes these interactions to determine both treatment success and adverse effects. By bridging mechanistic insights with clinical evidence, this Research Topic highlights the potential of host-targeted and biomarker-guided strategies to refine interferon-based therapies and advance more effective and better-tolerated approaches for the management of chronic hepatitis B.
